# Application and Reliability of Accelerometer-Based Arm Use Intensities in the Free-Living Environment for Manual Wheelchair Users and Able-Bodied Individuals

**DOI:** 10.3390/s21041236

**Published:** 2021-02-10

**Authors:** Brianna M. Goodwin, Omid Jahanian, Meegan G. Van Straaten, Emma Fortune, Stefan I. Madansingh, Beth A. Cloud-Biebl, Kristin D. Zhao, Melissa M. Morrow

**Affiliations:** 1 Health Sciences Research and Kern Center for the Science of Health Care Delivery, Mayo Clinic, Rochester, MN 55905, USA; briannagoodwin10@gmail.com (B.M.G.); Jahanian.Omid@mayo.edu (O.J.); VanStraaten.Meegan@mayo.edu (M.G.V.S.); Fortune.Emma@mayo.edu (E.F.); 2 Assistive and Restorative Technology Laboratory, Rehabilitation Medicine Research Center, Physical Medicine and Rehabilitation, Mayo Clinic, Rochester, MN 55902, USA; Madansingh.Stefan@mayo.edu (S.I.M.); Zhao.Kristin@mayo.edu (K.D.Z.); 3 Program in Physical Therapy, Mayo Clinic School of Health Sciences, Rochester, MN 55902, USA; Cloud.Beth@mayo.edu

**Keywords:** spinal cord injury, inertial measurement units, wearable sensors, upper extremity, free-living data collection

## Abstract

Arm use in manual wheelchair (MWC) users is characterized by a combination of overuse and a sedentary lifestyle. This study aimed to describe the percentage of daily time MWC users and able-bodied individuals spend in each arm use intensity level utilizing accelerometers. Arm use intensity levels of the upper arms were defined as stationary, low, mid, and high from the signal magnitude area (SMA) of the segment accelerations based on in-lab MWC activities performed by eight MWC users. Accelerometry data were collected in the free-living environments from forty MWC users and 40 sex- and age-matched able-bodied individuals. The SMA intensity levels were applied to the free-living data and the percentage of time spent in each level was calculated. The SMA intensity levels were defined as, stationary: ≤0.67 g, low: 0.671–3.27 g, mid: 3.27–5.87 g, and high: >5.871 g. The dominant arm of both MWC users and able-bodied individuals was stationary for most of the day and less than one percent of the day was spent in high intensity arm activities. Increased MWC user age correlated with increased stationary arm time (R = 0.368, *p* = 0.019). Five and eight days of data are needed from MWC users and able-bodied individuals, respectively, to achieve reliable representation of their daily arm use intensities.

## 1. Introduction

The majority of individuals with spinal cord injury (SCI) are non-ambulatory and require a wheelchair for their daily mobility [1]. Individuals with SCI who use a manual wheelchair (MWC users) have a high prevalence of musculoskeletal pain and injury [2,3,4] and the shoulder is the most common site of this pain and pathology [5,6]. Further, greater than 15% of individuals with acquired SCI reported shoulder pain as “unbearable” [3] and chronic MWC users are reported to have a four times higher prevalence of rotator cuff tears compared to age-matched able-bodied individuals [2]. The upper extremity is essential for MWC users as it is used for both mobility and activities of daily living (ADLs). Monitoring how the arms are used in the free-living environment may lead to useful information about mechanisms of injury and different use patterns between MWC users and able-bodied individuals. These insights could lead to improved shoulder preservation guidelines for MWC users. 

There are multiple contributing theories of shoulder pathology development for MWC users, including arm overuse and a sedentary lifestyle. Specifically, the load bearing nature of transfers and the repetitive task of propulsion are thought to lead to overuse of the arms for MWC users [7]. Overuse of the arms during MWC-based activities (mobility and ADLs) can contribute to increased pain and pathology in the shoulder for this population [8,9]. However, the etiology of shoulder pain and pathology is multifactorial and is not due to arm overuse alone [10]. Individuals with SCI are also overall more sedentary than able-bodied individuals, which may lead to a decrease in the overall use of the arms for MWC users compared to the able-bodied population [11,12]. The contradiction of a more sedentary lifestyle and elements of arm overuse complicate the understanding of the link between the intensity of arm use of MWC users and the associated shoulder pain and pathology. 

Wearable sensors such as accelerometers and inertial measurement units (IMUs) are low cost, accurate tools that have been used for monitoring and quantifying physical activity and clinical motion analysis in the able-bodied population and individuals with neurological conditions [13,14,15]. A few groups have created and validated overall energy expenditure or activity level thresholds specific for manual wheelchair users with wrist worn accelerometers [16,17]. Additionally, metrics from accelerometers placed on the upper arm during propulsion [18] and a variety of other functional activities [19] have been used to estimate physical activity levels. Although these methods are useful for estimating overall physical activity levels for individuals with SCI, they have not been used to understand the intensity of arm use, as a measure of overuse. Further, the reliability of these methods when applied to the free-living environment is largely unknown. The levels of activity and arm use vary from day to day within subjects due to environmental factors, individual characteristics, variations in daily schedules, and health condition. Therefore, it is important to investigate the number of days of monitoring that are needed to obtain a reliable representation of the overall arm use of a subject. A holistic daily view of the intensity of arm use in MWC users compared to able-bodied individuals may uncover patterns which provide context to understanding the increase in pain and pathology for MWC users. 

This study aimed to define and explore arm use intensity levels (stationary, low, mid, and high) as measured with IMUs worn on the upper arms. We defined intensity of arm use as an acceleration-based measure of the magnitude of upper-arm movement calculated from the signal magnitude area (SMA). Intensity, as described in this paper, should not be compared to intensity of physical activity related to energy expenditure or the rate of perceived exertion. Although we captured data with an IMU that contained an accelerometer and gyroscope, we only utilize the acceleration data. We are interested in field-based study applications wherein the participant is not required to charge the sensors every night which is required when using a gyroscope in the field. We report the preliminary results for the percentage of daily time MWC users and able-bodied individuals spend in each arm use intensity level throughout a typical day in the free-living environment, explore the effects of pain, time since injury, and age, and describe the reliability of the metrics for both cohorts. 

## 2. Methods

### 2.1. Study Design

**Figure 1:**
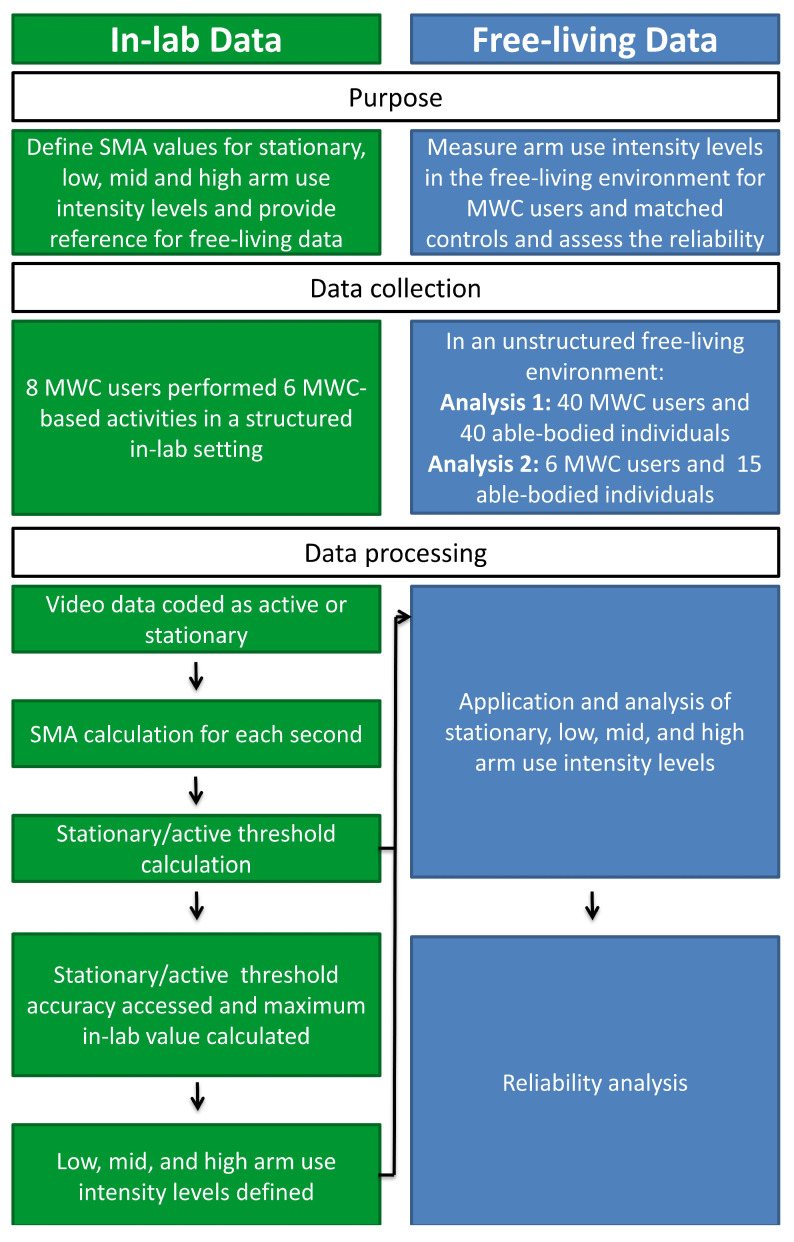
The purpose, data collection, and data processing flow of defining signal magnitude area (SMA) arm use intensity levels for the in-lab and free-living data utilized in this study.

Two separate data collections were employed to (1) define the arm use intensity levels and (2) estimate the percentage of daily time MWC users and able-bodied individuals spend in each arm use intensity level and test the reliabilities (Figure 1). First, in-lab IMU and video data, previously collected from a sample of MWC users with SCI [20], were utilized to determine the range of SMA magnitudes that defined each of the arm use intensity levels (stationary, low, mid, and high). After classification accuracy was assessed for the stationary and active threshold, the in-lab levels were applied to IMU data collected in the free-living environment from a different sample of MWC users with SCI and sex and age-matched able-bodied individuals. The percentage of time spent in each arm use intensity level was calculated. Additionally, the single-day reliabilities of all arm use intensity level metrics were calculated from an un-matched subset of both SCI and able-bodied individuals and used to estimate the required number of monitoring days needed to achieve a reliable representation of daily arm use intensity levels for both cohorts. 

All aspects of the study were approved by Mayo Clinic Institutional Review Board (IRB 15-004974, primary approval date: 07 September 2015). Individuals with SCI who were 18–70 years of age and using a MWC as their main mode of mobility for at least one year were recruited for the in-lab and free-living field data collections through querying medical databases and care providers of local clinics. In-lab inclusion criteria included active shoulder range of motion needed to complete the lab wheelchair-based lab activities (~150° of humeral elevation). Sex- and age- (±2.5 years) matched able-bodied individuals were recruited to participate in the free-living data collections. Participants were excluded in all parts of the study if they had prior significant surgery or injury to the shoulder or had cognitive impairments which may have limited their ability to follow instructions and adhere to the protocol. Additionally, participants were excluded from the free-living data collections if they had previous diagnosis of a complete supraspinatus tear or were unable to receive an MRI of one or both shoulders as the data presented here is part of a larger longitudinal study following the natural history of the rotator cuff.

### 2.2. In-Lab Data Collection for Intensity Level Calculation

**Figure 2:**
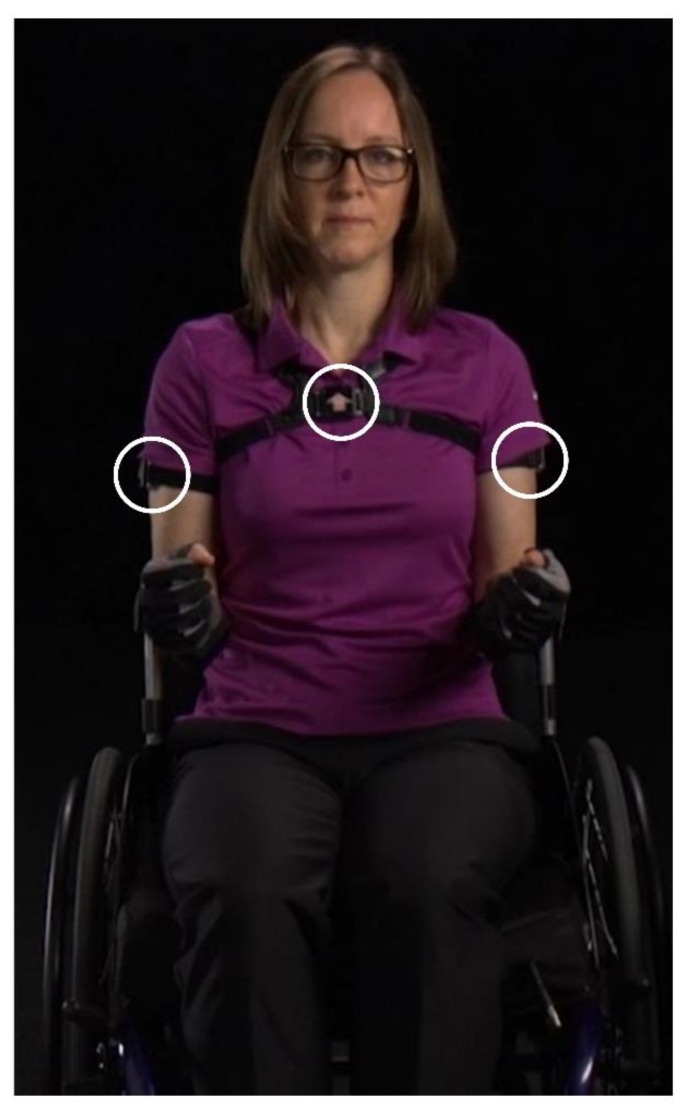
Three inertial measurement units (IMU) were secured to all participants on both of their lateral arms and their chest using elastic straps. The circles indicate where the IMUs are attached. Note: The individual pictured is study staff and not a research participant.

IMU and video data were collected while participants performed six MWC-based activities as part of an ancillary study [20,21]. Two IMUs were secured with straps to each of the participant’s upper arms (Emerald, APDM, Inc., Portland, OR, USA) and data were collected at 128 Hz (Figure 2). Video data were collected at 60 Hz using a handheld digital camera throughout the entire data collection. Participants were asked to complete six MWC-based activities: (1) counter height reaching (36 inches above the ground), (2) overhead reaching (54 inches above the ground), (3) 6.8 kg cross-body lifting of a backpack from the floor on the side of his/her wheelchair to a plinth on the other side, (4) level transfers between the MWC and plinth, (5) level MWC propulsion (on rollers), and (6) 5° incline MWC propulsion (on rollers with a wooden board under the casters to create an incline). During the reaching activities, each participant retrieved and returned an aluminum (soup) can (0.45 kg) from a table (counter height) or shelf (overhead). For reaching and cross-body lifting, each participant used the arm that they self-selected as their trailing limb during their daily car transfers into the car or the side to which they transfer most frequently. Additionally, participants self-selected their cadence during propulsion. 

Participants were asked to perform 10 reaches for both counter height and overhead reaching, three cross body lifts (from ground to plinth and back to ground), and six transfers (from MWC to plinth or plinth to MWC). They were additionally asked to perform two MWC propulsion trials: propulsion on level rollers for approximately two minutes and propulsion on a simulated incline condition for approximately 15 s. In cases where technical difficulties occurred, additional trials were performed, if possible. In some cases, fewer numbers of trials or shorter duration of MWC propulsion were performed based on the participant’s physical capacity. On average participants completed 11 ± 3 counter height reaches, 11 ± 3 overhead reaches, 4 ± 2 cross-body lifts, 6 ± 2 transfers, 110 ± 51 s of level propulsion, and 25 ± 5 s of inclined propulsion. The activities and time naturally occurring between them were included in the data used to define arm use intensity levels to ensure inclusion of natural resting periods.

### 2.3. Free-Living Data Collection 

Similar to the in-lab data collection, participants were fit with two IMUs on their bilateral arms (Emerald or Opal, APDM, Inc.). Participants were asked to wear the sensors for at least eight hours during a short or extended data collection period. During the short collection period one or two days were required. For the extended data collection period, a subset of both MWC and able-bodied participants were asked to wear the sensors for four consecutive days (one weekend day and three weekdays). The extended data collection was used for the reliability analysis. 

Participants charged the sensors each night and were instructed to not change their regular activities. Each participant was provided in-person, written, and video instructions to increase protocol adherence. Participants were provided a pre-paid envelope or met study staff in person to return the sensors after completion of the data collection. Participants in the MWC cohort completed the Wheelchair User Shoulder Pain Index (WUSPI) for both right and left arms. Although we acknowledge that the WUSPI was designed to be filled out once, as part of a larger study, the WUSPI was filled out for both arms to evaluate pain and function as it related to each arm. To complete the WUSPI, participants rated their shoulder pain when completing 15 functional activities on a visual analog 10 cm scale between “no pain” (0 cm) and “worst pain ever experienced” (10 cm) [22]. Possible overall raw WUSPI scores ranged between 0 (no pain) and 150 (worst pain ever experienced in all 15 items). The WUSPI has been shown to be valid and reliable for this population [23]. For the individuals who did not perform certain functions, the performance-corrected WUSPI score (PC-WUSPI) was calculated by dividing the raw WUSPI score by the number of items completed and multiplying by 15. 

### 2.4. Data Processing

Raw acceleration data from the IMUs were downloaded through Motion Studio (APDM, Inc., Portland, OR, USA) for both the in-lab and free-living data collections. The linear acceleration data were used to quantify the intensity of arm use by calculating the SMA with a custom MATLAB (Mathworks, Natick, MA, USA) code. SMA is a measure of the intensity of movement over one second; the methods are described elsewhere [24]. In short, the acceleration signal was filtered with a centered median filter to reduce noise spikes (window size of three frames) [24,25]. The gravitational component of the signal was then calculated by using a third-order zero phase lag elliptical low pass filter with 0.25 Hz cut-off frequency, 0.01 dB passband ripple and −100 dB stopband ripple. The gravitational component of the acceleration signal was subtracted from the original signal to leave the gravitational component due to body movement and the SMA was calculated for each second of data [24].

### 2.5. Arm Use Intensity Level Definitions (In-Lab Data)

VLC media player (VideoLAN Organization, Paris, France) was used to view all in-lab video data for analyses. For the six in-lab MWC-based activities and the time naturally occurring between them, one rater (EF) with more than eight years of movement and video analysis experience coded each second of data as either stationary or active [20]. A threshold between stationary and active arm use was calculated from a receiver operating characteristic (ROC) curve; the SMA value which maximized specificity and sensitivity for a subset of data (6 of 8 participants, ~78% of total data) was chosen as the threshold (0.67 g). The remaining data (2 of 8 participants, ~22% of total data) were lumped together and used assess the accuracy of this threshold.

The low, mid, and high arm use levels were then defined. The maximum in-lab SMA values from all participants were averaged (8.46 ± 1.59 g). For the dominant arm, the maximum SMA was reached during incline propulsion for four participants and during transfers for four participants. Three evenly spaced intensity intervals were then defined between the maximum value of active movement and the minimum value of active movement (active/stationary threshold), yielding low, mid, and high intensity levels. SMA analyses of the active portions of the in-lab MWC-based activities provide a reference for the free-living SMA data. Therefore, the mean, maximum and minimum active SMA values were calculated for each of the six in-lab MWC-based activities for each participant.

### 2.6. Arm Use Intensity Level Application to Free-Living Data

Free-living data were excluded if less than eight hours of useable data were collected for each day (after elimination of non-wear time). All data were visually inspected to ensure non-wear time was eliminated. Eight hours was chosen as the minimum collection period for a full day as a balance between sensor battery life and inclusion of data. Utilizing the arm use intensity levels from the in-lab data, the daily percentages of time that individuals spent in each intensity level were calculated for each participant’s dominant arm. If more than one day existed, the average time across the two days of data was calculated for each participant and the two cohorts. The correlation between age (both cohorts) and pain (PC-WUPSI score) for the SCI cohort were calculated. Similar methods were used to calculate the average percentage of time in each arm use intensity level for the reliability analysis. 

### 2.7. Statistical Analysis

Statistical analyses were performed in SPSS 25 (IBM Corp., Armonk, NY, USA). Non-parametric tests were used due to the non-homogeneity in participant demographics (age, sex, type of SCI (complete/incomplete), and level of SCI) and visually inspected non-normal distribution of percentages in each intensity level. Separate Kruskal-Wallis tests were used to test the effect of cohort. When significant differences were observed, post-hoc analyses were completed using Wilcoxon Signed Ranks test. The effect size was calculated as the ratio of the z-value to the square root of the number of participants. Additionally, Spearman’s correlation was used to investigate the association between age, pain (measured by the PC-WUSPI), and time since injury with the percentage of time the MWC cohort spent in each arm use intensity. P-values less than 0.05 were considered statistically significant in all tests.

For the reliability analysis, single-day reliabilities of arm use intensity levels were calculated based on the weekdays and weekends (four days: one weekend and three weekdays) measurements and only weekday (three weekdays) measurements, using reliability analyses in SPSS 25. When available weekday and weekend measurements were consecutive days from Sunday through Wednesday and only weekday measurements were consecutive days from Monday through Wednesday. Single-day reliability was defined as single measure Intraclass Correlation Coefficients (ICC), calculated based on one-way random effects model [15,26]. For both cohorts the required days of monitoring needed to achieve moderate, good, and excellent reliabilities were calculated using the Spearman-Brown prophecy formula based on the weekday and weekday and weekend measurements separately [27]. Reliability coefficient values between 0.5 and 0.75 are considered as moderate, 0.75 and 0.9 as good, and higher than 0.9 as excellent reliability [28]. For all analyses, statistical significance was set at an alpha level of *p* < 0.05.

## 3. Results

### 3.1. Arm Use Intensity Levels during In-Lab MWC-Based Activities 

Eight participants were included in the in-lab data collection (Table 1). Six of the eight participants were used to calculate the threshold between stationary and active arm use; the remaining two participants were used to assess the accuracy of the threshold. The active/stationary threshold had specificity, sensitivity, and overall accuracy values of 94%, 80%, and 89%, respectively. The SMA ranges for each level are as follows: stationary (SMA ≤ 0.67 g), low intensity (0.67 g < SMA ≤ 3.27 g), mid intensity (3.27 g < SMA ≤ 5.87 g), and high intensity (SMA > 5.87 g). 

**Figure 3:**
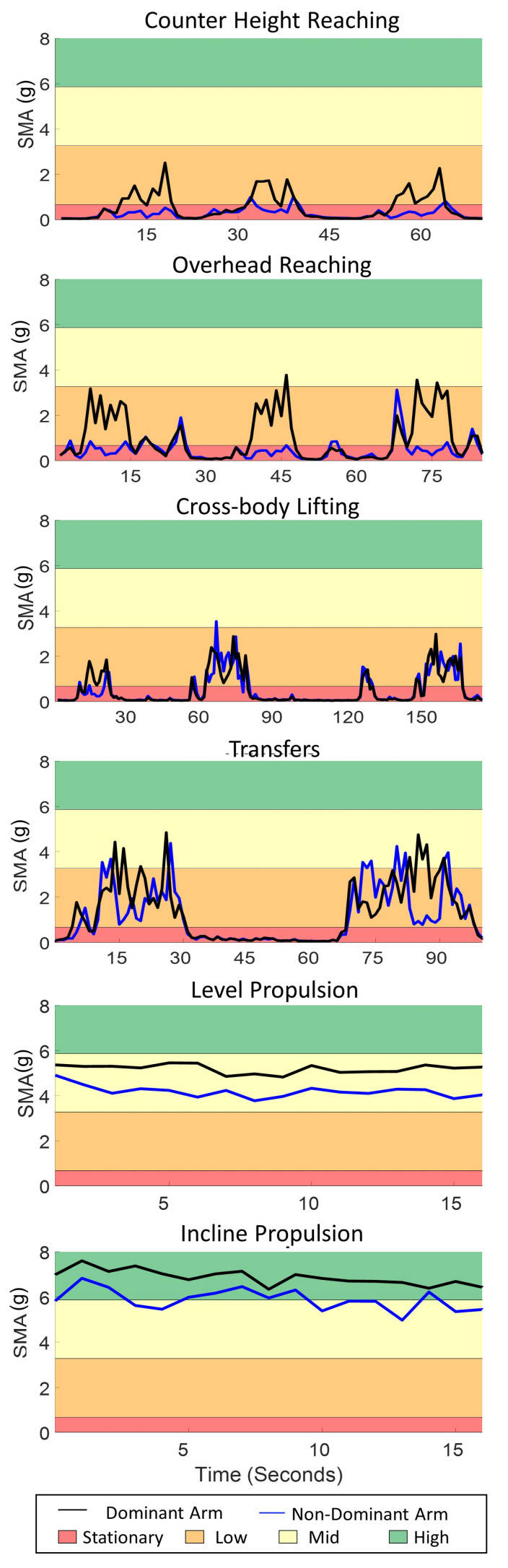
Representative signal magnitude area (SMA) data from one participant completing three counter height reaches, three overhead reaches, three cross body lifts, two transfers, level propulsion and incline propulsion in a structured lab setting for their dominant (black line) and non-dominant (blue line). The shaded areas represent the am use intensity levels (red: stationary, orange: low, yellow: mid, green: high).

Representative data from one participant completing all activities are shown in Figure 3. The mean, maximum and minimum SMA for each activity give context to the arm use intensity levels participants utilized to accomplish MWC-based activities in the lab (Table 2). 

### 3.2. Free-Living Data: Percentage of the Day in Each Arm Use Intensity Level

Forty MWC users and 40 sex- and age- (±2.5 years) matched controls were enrolled in the matched analysis (Table 1). Data were collected for an average of 11.5 ± 2.3 h per day for the MWC cohort and 11.3 ± 2.1 h per day for the control cohort. 92.5% of the participants in the control cohort had two days of usable data, while only 75% of the MWC participants collected two days of useable data. The second day of control data were excluded for three participants because the sensors were not worn on day two (1 participant) and less than eight hours of data were collected on the second day (2 participants). Additionally, the second day of data were excluded for the MWC cohort for 10 participants because the sensors malfunctioned (2 participants), the sensors were not worn on day two (3 participants), and less than eight hours of data were collected on the second day (5 participants). 

The results from the Kruskal-Wallis test indicated that there was a significant effect of cohort (MWC or control) on the percentage of time spent in arm use intensity levels (*p* < 0.05). Post hoc tests revealed, that although low and high intensity levels were similar between cohorts, the MWC cohort spent significantly less time in the mid intensity level for both arms (Table 3). The MWC cohort also trended toward spending more time in stationary than matched controls (~8% or ~50 min more per day). 

**Figure 4:**
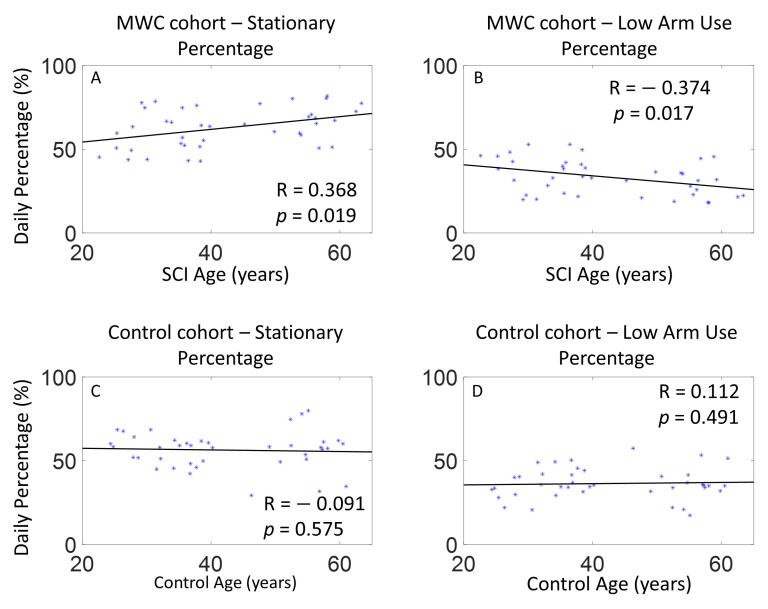
The effect of age on the percentage of daily time spent stationary and in the low arm use intensity levels for the MWC and control cohorts on the dominant arm: (**A**) MWC stationary, (**B**) MWC low arm use intensity level, (**C**) control stationary, (**D**) control low arm use intensity level.

The Spearman correlation analyses indicated that as the age of the MWC cohort increased, the time spent stationary also increased (Figure 4). Additionally, younger MWC users spent a significantly higher percentage of time in the low arm use intensity level compared with older MWC users. Age did not have a significant effect on mid and high intensity levels and this trend was not observed for the controls.

On the dominant side of the MWC cohort, increased PC-WUSPI scores were significantly associated with less time in high arm use intensity level (*p* = 0.012, R = −0.395). There were no other effects of the PC-WUSPI or time since injury. 

### 3.3. Reliability of Arm Use Levels 

A subset of six MWC users and 15 able-bodied individuals (unmatched) were enrolled in the reliability analysis (Table 1). Data were collected on average for 9.7 ± 0.8 and 10.3 ± 1.2 h for the MWC and able-bodied cohorts, respectively. All MWC users and 73% (11/15) of able-bodied individuals had consecutive weekday & weekend measurements from Sunday to Wednesday and only weekday measurements from Monday to Wednesday. When consecutive days could not be achieved (4/15 able-bodied individuals), data from three non-consecutive weekdays and one weekend day were used in analysis. Single-day reliabilities of arm use intensity levels ranged from moderate to poor for both MWC and able-bodied cohorts (Table 4). The results from the weekday & weekend measurements indicated that a good reliability (>0.75) is reached when monitoring MWC users for five days and able-bodied individuals for eight days. The results from only weekday measurements indicated that a good reliability (>0.75) is reached when monitoring MWC users for three weekdays and able-bodied individuals for four weekdays (Table 4). 

## 4. Discussion

The primary purpose of this study was to explore the application of arm use intensity levels (stationary, low, mid, and high) measures derived from the acceleration of the upper arms and estimate the percentage of time the dominant arm of MWC user and able-bodied individuals spend in each arm use intensity level. We also aimed to explore the relationship with age, PC-WUSPI score, and calculate the single-day reliability of the metrics and estimate the number of days which are required to obtain a reliable representation of overall daily arm use intensities. The results indicated the dominant arm of both cohorts was stationary and in low arm use intensity levels for the majority of the day and the dominant arm of older MWC users was more stationary. To achieve good reliabilities for all arm use intensity levels throughout an entire week (weekdays and weekend days) at least five and eight days of data are needed for MWC users and able-bodied individuals, respectively. 

Although specific activities were not identified in the free-living environment in this study, the in-lab MWC-based activities help give context to the measured arm use intensity levels during free-living. For example, the data presented here suggests that level and incline propulsion occur in the mid and high arm use levels. Additionally, other in-lab wheelchair-based activities achieve multiple arm use intensity levels; for instance, during transfers arm use intensities range from stationary to high levels. It is critical to recognize that the arm use metrics are only based off acceleration values and no load was measured. The load bearing nature of transfers is thought to contribute to arm overuse for MWC users and increase the risk of secondary upper extremity injury in this population [7]. Further, the humeral elevation of the arms was not measured in this study. The combination of the second-by-second arm use intensity levels with the humeral elevation would provide a more holistic view of the way the arms are used and could potentially shed more light on the mechanisms of increased rotator cuff pathology.

The able-bodied individuals trended toward spending a larger percentage of time in the mid arm use intensity level than MWC users. Based on the lab data, level propulsion, inclined propulsion, transfers, overhead reaching, and cross body lifting had portions of the activity that achieved a mid-intensity level. If lab-based arm use intensities are comparable to free-living arm use intensities, we may conjecture that the able-bodied cohort performs activities in the mid arm use intensity level more frequently than MWC users. However, we did not collect reference activities for the able-bodied cohort, so the comparison across intensities is meant as an initial description into characterizing how MWC users and able-bodied adults may differ in how they use their arms throughout a typical day. 

The MWC and able-bodied cohorts spent most of their day with their arms stationary. Although, some of this time likely includes rest and recovery of the musculoskeletal system of the arms, likely some of this time does not allow for recovery. Industrial ergonomic studies have shown that short periods of rests (less than 5 s) do not provide an adequate recovery time during repetitive upper extremity tasks [29,30]. The SMA was defined in one second epochs; therefore, it is possible that active arm use occurred immediately before and after stationary seconds. The incorporation of the duration of stationary levels would aid in this interpretation. Additionally, ergonomics literature has reported that for every 10 min of work a 90 s rest is required [31]; thus, it will be interesting to understanding whether MWC users achieve the recommended rest to work ratios and how this correlates to pathology development among MWC users.

There was a significant effect of age on arm use intensity levels in the MWC cohort suggesting that older MWC users spend more time with their dominant arm stationary and younger MWC users spend more time in the low arm use intensity level compared to older users. While studies of able-bodied individuals have measured activity levels at the trunk, our data on arm use follow similar patterns related to age and activity for the MWC cohort. Studies on able-bodied individuals suggest that individuals over the age of 60 spend 65–80% of their waking day in sedentary behavior [32]; further, accelerometry data has shown that individuals age 70–85 are more sedentary than other age groups [33]. There was only one matched pair over 60 years old in the current study; therefore, MWC users may exhibit stationary arm use earlier in life than the able-bodied population. Further research with larger sample sizes of older participants is needed to confirm this finding.

While high intensity arm uses only accounted for 1% of the total day (~4 min per day), increased pain of the dominant shoulder (PC-WUSPI score) in MWC users was associated with decreased time in the high arm use intensity level. Due to the limited sample size and very small portion of the day that includes high levels of arm use, caution should be used when drawing conclusions about this finding. While high intensity use could be avoided in order to decrease pain, exercise in the form of high intensity use could be protective against developing pain. Further investigation is needed to understand the clinical implications of this potential finding.

The results from the reliability analysis indicate that at least five and eight days of data are needed from the MWC users and able-bodied individuals, respectively, to achieve reliable representation of their overall daily arm use intensity throughout a week (weekdays and weekends). However, only four and three days of data are needed to obtain a reliability representation of only weekdays for MWC users and able-bodied individuals, respectively. The difference between the weekday & weekend and only weekday analysis is primarily due to a difference in arm use intensity levels during weekdays versus weekends. One other study has investigated the reliability of wheelchair-based metrics from wrist-worn accelerometry data for individuals with SCI who use MWCs [26]. The metrics included measures of physical activity intensity, wheeling quantity, and movement quality from data collected during in- and out-patient settings. Their results suggest that four days of data are needed for good reliability of movement quality and three days are required for most other metrics in the out-patient setting [26]. Schneider et al. found no difference between weekdays and weekends in wheelchair distance traveled [26]. This difference between the current study and Schneider et al. could be in part due to a difference in metrics measured, time since injury, or other differences in study design. The results presented here included individuals on average 20+ years since injury, while Schneider et al. included participants 300–400 days post injury [26]. 

It is important to understand the study limitations when interpreting our findings. First, no angular kinematic data or information about when the arm was loaded (push phase of propulsion, lifting an object, transfers, etc.) were included in these analyses. Understanding both the load and arm position during each arm use intensity level would allow more insight into the pathomechanics of injury for MWC users and how it differs from able-bodied individuals. Implementing algorithms which calculate and integrate arm loading and the position of the arm with the arm use intensity levels were out of the scope of the current study; however, analysis of the arm position during arm use intensity levels is an active area of research for our group. Additionally, the arm use intensity levels were defined based on a small cohort of MWC users and only six in-lab activities. A larger cohort of MWC users may perform activities differently and activities may be performed at higher SMA levels in the free-living environment. Further, able-bodied individuals may perform ADLs with different arm use intensity levels than the MWC cohort; however, the use of a single set of levels allowed for exploration and a descriptive comparison between both cohorts. Finally, the reliability analysis in this study demonstrated that five and eight days of data are needed from MWC users and able-bodied individuals, respectively, to achieve reliable representation of their daily arm use intensities; however, due to participant availability, only one or two days of free-living data were collected for the participants. Caution should be taken when drawing conclusions from differences between the arm use intensity levels of the MWC and able-bodied cohorts. Further studies will include more days of IMU data collection. 

## 5. Conclusions

This study aimed to define arm use intensity levels utilizing IMUs worn on the upper arms, apply intensity levels to free-living data of MWC users and able-bodied individuals, and measure the number of days of data needed to achieve a reliable representation of arm use. Overall, MWC users and able-bodied individuals spent over half the day with their arms stationary. The MWC and able-bodied cohorts spent less than 4% (~30 min) and 7.3% (~50 min) of the day in and above mid arm use intensity levels, respectively. Increased MWC user age correlated with increased stationary arm time. Five and eight days of data are needed to achieve good reliabilities for all arm use levels throughout weekdays and weekends for MWC users and able-bodied individuals, respectively. Understanding the intensity levels of arm use for MWC users compared with able-bodied individuals may aid in understanding the mechanisms of rotator cuff pathology which occur more frequently in MWC users than able-bodied individuals. The causes of increased shoulder pain and pathology for MWC users are multifactorial and the intensities of arm use are only one piece of a complicated puzzle. Other characteristics of use, such as duration of arm use bouts, amount of rest/recovery between repetitive motions, over-head humeral elevation and loading contribute to pain and pathology. Future work from our group is focused on understanding factors of arm overuse of MWC users and the relationship between overuse and the progression of shoulder pain and pathology utilizing IMUs for data collection in the free-living environment.

## Figures and Tables

**Table 1:** In-lab and free-living (Manual Wheelchair (MWC) and able-bodied individuals) participant demographics. M = Males, F = Females.

	In-Lab	MWC (Comparison Analysis)	Able-Bodied (Comparison Analysis)	MWC (Reliability Analysis)	Able-Bodied (Reliability Analysis)
Participants (n)	8	40	40	6	15
Age (years), mean + SD (range)	39.4 ± 12.6 (23–61)	42.4 ± 12.2 (23–63)	42.3 ± 11.9 (24–61)	51.5 ± 9.6 (31–59)	42.2 ± 12.0 (25–59)
Sex	7 M/1 F	32 M/8 F	32 M/8 F	4 M/2 F	12 M/3 F
Injury Level (n)			-		-
C6-C7	4	7		1	
T1-T8	4	18		1	
T9-L1	0	15		4	
Time since injury (years), mean ± SD (range)	9.6 ± 11.3 (1–33)	11.5 ± 11.5 (1–39)	-	21.9 ± 13.1 (2–35)	-
Pain (n)	5	26	10	5	2

**Table 2:** The mean ± standard deviation of the minimum, maximum, and mean signal magnitude area (SMA) for the eight participants who completed the six in-lab manual wheelchair-based activities.

		Minimum SMA (g)	Maximum SMA (g)	Mean SMA (g)
Counter height reaching	Reaching arm	0.5 ± 0.2	2.6 ± 0.4	1.2 ± 0.3
	Non-reaching arm	0.2 ± 0.2	2.0 ± 1.0	0.7 ± 0.5
Overhead reaching	Reaching arm	0.6 ± 0.2	4.1 ± 0.7	2.0 ± 0.2
	Non-reaching arm	0.2 ± 0.1	3.4 ± 1.8	1.1 ± 0.5
Cross-body lifts	Dominant	0.3 ± 0.2	5.0 ± 0.9	1.7 ± 0.3
	Non-Dominant	0.3 ± 0.1	4.8 ± 0.9	1.6 ± 0.2
Transfers	Dominant	0.3 ± 0.2	7.5 ± 1.2	2.3 ± 0.4
	Non-Dominant	0.2 ± 0.1	8.1 ± 1.7	2.2 ± 0.4
Level propulsion	Dominant	1.0 ± 0.3	7.1 ± 1.4	5.4 ± 1.2
	Non-Dominant	1.0 ± 0.4	6.8 ± 2.0	5.0 ± 1.4
Incline propulsion	Dominant	1.0 ± 0.5	7.4 ± 1.6	5.3 ± 1.0
	Non-Dominant	1.0 ± 0.6	7.1 ± 1.9	5.0 ± 1.1

**Table 3:** The defined in-lab signal magnitude area (SMA) range for each arm use intensity level and the mean ± standard deviation percentage of the day the dominant arm of the manual wheelchair users (MWC) and able-bodied individuals spent in each arm use level in the free-living environment.

Arm Use Intensity Level	SMA Range (g)	MWC (% of day)	Able-Bodied (% of day)	*p*-Value ^1^	z-Value	Effect Size
Stationary	SMA ≤ 0.67	62.8 ± 11.7	56.4 ± 10.8	0.055	−1.922	−0.215
Low intensity	0.67 < SMA ≤ 3.27	33.3 ± 10.3	36.4 ± 9.1	0.270	−1.102	−0.123
Mid intensity	3.27 < SMA ≤ 5.87	3.4 ± 1.9	6.7 ± 2.7	<0.001 *	−4.731	−0.529
High intensity	SMA > 5.87	0.6 ± 0.6	0.6 ± 0.5	0.936	−0.081	−0.009

^1^ Wilcoxon Signed Ranks Test. * Indicates statistical significance (*p* < 0.05).

**Table 4:** Intraclass reliability coefficients (ICCs) for MWC and able-bodied cohorts based on only weekday measurements (3 days) and weekday & weekend (3 weekdays, 1 weekend) measurements and the number of required days of monitoring to obtain moderate, good, and excellent reliabilities.

	Single Day Reliability	F Test	Days of Monitoring to Achieve Reliabilities of:		
	ICCs	Sig	0.5	0.75	0.9
**MWC Cohort–Only Weekdays**					
**Stationary**	0.620	0.006	0.61	1.84	5.52
**Low**	0.587	0.009	0.70	2.11	6.33
**Mid**	0.454	0.035	1.20	3.61	10.82
**High**	0.487	0.026	1.05	3.16	9.48
**MWC Cohort–Weekdays & Weekend**					
**Stationary**	0.441	0.011	1.27	3.80	11.41
**Low**	0.413	0.016	1.42	4.26	12.79
**Mid**	0.389	0.021	1.57	4.71	14.14
**High**	0.444	0.011	1.25	3.76	11.27
**Able-bodied Cohort–Only Weekdays**					
**Stationary**	0.638	0.001	0.57	1.70	5.11
**Low**	0.722	0.001	0.39	1.16	3.47
**Mid**	0.591	0.001	0.69	2.08	6.23
**High**	0.567	0.001	0.76	2.29	6.87
**Able-bodied Cohort–Weekdays & Weekend**					
**Stationary**	0.543	0.001	0.84	2.52	7.57
**Low**	0.567	0.001	0.76	2.29	6.87
**Mid**	0.292	0.007	2.42	7.27	21.82
**High**	0.646	0.001	0.55	1.64	4.93

## Data Availability

The data presented in this study are available on request from the corresponding author. The data are not publicly available due to the ongoing nature of the longitudinal study.
